# Long-Term Organic Fertilization Drives Soil Organic Carbon Accumulation in Black Soil Through Microbial Necromass Carbon and CAZyme-Mediated Carbon Turnover

**DOI:** 10.3390/microorganisms14091970

**Published:** 2026-09-07

**Authors:** Yang Liu, Haoyan Li, Xinxin Guo, Nan Wang, Ning Huang, Hongbin Wang, Jinhua Liu, Chenyu Zhao, Luze Yang, Biao Sui, Xingmin Zhao

**Affiliations:** 1 Key Laboratory of Saline-Alkali Soil Reclamation and Utilization in Northeast China, Ministry of Agriculture and Rural Affairs, Jilin Provincial Key Laboratory of Saline-Alkali Soil Reclamation and Utilization, Jilin Agricultural University, Changchun 130118, China; 2 Faculty of Engineering and Green Technology, Universiti Tunku Abdul Rahman, Kampar 31900, Malaysia; guox@utar.edu.my

**Keywords:** carbon sequestration, black soil, straw return, manure application

## Abstract

Long-term organic inputs increase soil organic carbon (SOC) in black soil; however, it remains uncertain if straw and manure enhance SOC via separate microbial necromass carbon (MNC) routes. Therefore, a 12-year field experiment was conducted to explore this mechanism. Treatments included inorganic fertilizer, partial substitution of inorganic fertilizer with straw, and partial substitution of inorganic fertilizer with manure, along with a no-fertilizer control. According to the results, compared with inorganic fertilizer alone, treatments involving both organic manure and straw returned markedly raised the levels of SOC, MNC, bacterial necromass carbon (BNC), and fungal necromass carbon (FNC). Notably, compared to the CK treatment, N75S showed a greater contribution of MNC to SOC (MNC/SOC), but in both N75M and M, the MNC/SOC was lower than in CK. The CAZyme genes related to hemicellulose, lignin, chitin, and peptidoglycan differed among treatments. Elevated gene abundances for plant-derived lignin and hemicellulose decomposition were observed under M and N75M, whereas they reduced the genes responsible for peptidoglycan and glucan. However, under N75S, there was a notable rise in the number of genes responsible for hemicellulose and lignin, as well as an increase in genes that degrade chitin and peptidoglycans. In conclusion, this finding demonstrates that long-term straw return tends to promote SOC stabilization primarily through the microbial necromass pathway, while the application of manure over the long term tends to increase the total SOC primarily through the input of exogenous organic carbon.

## 1. Introduction

Black soil is known for its high humic substance content, deep soil layers, and abundant nutrient elements [1]. Northeast China belongs to one of the world’s top four black soil zones, serving as a critically important commercial grain production base in China, and making a major contribution to the preservation of national food security [2]. Black soil has lost approximately 50% of its organic matter over the last 40 years [3]. Microbial necromass carbon (MNC) consists of bacterial necromass carbon (BNC) and fungal necromass carbon (FNC), which has now been recognized as a major constituent of soil organic carbon (SOC). Based on comprehensive studies across multiple ecosystems, the contribution of MNC to SOC ranges from 30% to 60% [4]. MNC is characterized by sustained production, persistence in soil, and slow decomposition, thereby serving as a critical factor in the sustained capture of carbon [5,6]. Microbial carbon pump (MCP) theory describes two main pathways that lead to SOC formation: externally mediated modification through extracellular enzymatic activity, and internal turnover driven by microbial assimilation, biosynthesis and subsequent cell death [7]. Therefore, promoting MNC sequestration is critical for SOC sequestration strategies.

Soil extracellular enzymes primarily catalyze organic matter degradation through oxidation reactions and hydrolysis [8]. Soil microbes carry diverse functional genes responsible for producing extracellular enzymes [9]. CAZymes are essential for the degradation, modification, and synthesis of carbohydrates [10]. CAZyme composition and abundance serve as an indicator of the decomposition capacity of the soil microbial community’s capacity to decompose different organic compounds. CAZymes have been utilized to explore microbial responses to carbon cycling functions. A number of studies have revealed that certain auxiliary activity (AA) and glycoside hydrolase (GH) enzymes contribute to carbon cycling by degrading cellulose, chitin, and lignin, respectively [11,12]. Through correlation analyses and path modeling, Wang et al. [13] statistically linked CAZyme gene abundance with necromass carbon fractions and carbon pool components, thereby providing a testable mechanistic framework for the transformation pathway of “carbon-degradation genes → substrate decomposition → microbial carbon pump → necromass carbon accumulation”.

To investigate the variations in the activities of extracellular enzymes and CAZyme gene families related to carbon cycling, as well as their relationships with MNC, it is critical to understand how MNC contributes to SOC sequestration in agricultural systems.

Numerous recent studies have revealed that different fertilization treatments influence microbial community composition [14,15]. In agricultural systems, crop residue decomposition represents a major pathway of carbon input. The increased input of plant residues through straw return significantly influences the substrate utilization strategies of the soil microbial community [16,17]. Previous findings have indicated that manure application modifies the activity of carbon-fixing functional microbes, thereby increasing the abundance of soil carbon fixation genes [18]. Furthermore, numerous field studies have indicated that long-term application of organic manure effectively enhances carbon fixation gene abundance through altering the composition of the microbial community [19]. The use of organic fertilizer or chemical fertilizer significantly altered both the composition and functional potential of soil microbial communities associated with the carbon cycle, consequently impacting carbon fixation processes [20]. Earlier research has also identified a link between various fertilization methods and soil C dynamics in fluvo-aquatic soils [21]. In particular, Wang et al. [21] demonstrated that long-term straw return regulates C degradation genes through the combined effects of exogenous organic C input and nutrient optimization, thereby increasing SOC. In contrast, mineral fertilizer treatment upregulated genes involved in carbon fixation and organic matter decomposition by increasing plant biomass. However, a clear understanding of CAZyme characteristics in black soil is still lacking.

Earlier research has concentrated on the alterations in soil carbon pools under straw return and organic fertilizer application. However, limited studies have examined the influence of CAZyme-driven MNC accumulation on SOC pools. Consequently, drawing on a long-term black soil field trial, through metagenomic approaches, the community structure and functional potential of soil microbiomes were investigated. We investigated the content of MNC under different fertilization regimes and explored mechanisms underlying MNC accumulation, focusing on microbial community abundance, enzyme activity, and MCP efficiency. Based on the above background, we proposed the following four sequential mechanistic hypotheses: (1) Fertilization reshapes microbial community composition and CAZyme gene abundance; (2) upregulation of CAZyme genes drives enzyme activities and substrate decomposition; (3) enhanced MCP turnover promotes MNC accumulation; and (4) straw and manure operate through different MNC pathways as well as direct carbon input pathways.

## 2. Materials and Methods

### 2.1. Study Site Description

This long-term black soil field experiment is located in Chaoyangpo Town, Jilin Province (43°37′ N, 124°47′ E), northeast China, and began in 2013. This area experiences a moderate monsoon weather pattern, characterized by an average yearly temperature of 6.3 °C, yearly rainfall of 400 mm, and 127 days without frost. The management system included annual maize planting.

### 2.2. Long-Term Field Experiment Design

A randomized complete block design with three replications was adopted in this experiment. Each plot consisted of six ridges, each 15 m long and 3.6 m wide. Six treatments were established, including: (1) CK, no fertilizer; (2) PK, P_2_O_5_ 90 kg ha^−1^, K_2_O 100 kg ha^−1^; (3) NPK, N 180 kg ha^−1^, P_2_O_5_ 90 kg ha^−1^, K_2_O 100 kg ha^−1^; (4) N75S, N 135 kg ha^−1^, P_2_O_5_ 90 kg ha^−1^, K_2_O 100 kg ha^−1^, maize straw 11.7 t ha^−1^; (5) N75M, N 135 kg ha^−1^, P_2_O_5_ 90 kg ha^−1^, K_2_O 100 kg ha^−1^, cattle manure 12.2 t ha^−1^; and (6) M, only cattle manure 48.8 t ha^−1^. Maize was continuously cultivated under a spring maize monoculture system with a single-cropping regime per year. In the experiment, the chemical fertilizers utilized were urea (46% N, Changchun, China), diammonium hydrogen phosphate (46% P_2_O_5_, Changchun, China), calcium superphosphate (46% P_2_O_5_, Changchun, China), and potassium sulfate (60% K_2_O, Changchun, China), all purchased from the market. The straw used was maize straw (0.70% N, Changchun, China), and the organic manure was cattle manure (1.35% N, 1.09% P_2_O_5_, and 1.14% K_2_O, Changchun, China). Phosphorus and potassium fertilizers were applied as basal fertilizers once at the beginning of each growing season, repeated annually throughout the experimental period. The application of nitrogen fertilizer was divided into two segments: 40% for basal use and the rest for topdressing. Organic manure was applied entirely as a base fertilizer. Straw was crushed and directly applied to the soil surface. After harvest, crop residues were removed from the field. The N75S treatment received straw return according to the prescribed amount, while aboveground crop residues were removed in all other treatments.

### 2.3. Sample Collection

During April 2024, soil sampling from the 0–20 cm depth was conducted with the five-point method. For each plot, all collected subsamples were combined, homogenized to a uniform consistency, and immediately delivered to the laboratory. Visually apparent plant litter and stones were picked out. Each soil sample was allocated into three parts: the first was air-dried under cool, dry conditions for the measurements of amino sugars, SOC, and enzyme activities; the second was kept at 4 °C for microbial biomass carbon (MBC) determination; and the third was immediately stored at −80 °C for metagenomic analysis.

### 2.4. SOC, MBC and MNC

The K_2_Cr_2_O_7_ oxidation outer heating technique was used to quantify the SOC content.

The fumigation–extraction method was employed to determine MBC, following the procedure established by Vance et al. [22]. Fresh soil samples were fumigated with chloroform in a vacuum desiccator for 24 h, while control samples were incubated in the dark under the same temperature conditions for 24 h. Extraction of MBC was performed using K_2_SO_4_ solution, and the MBC content was calculated as the difference in carbon content between the fumigated and non-fumigated samples [22].

MNC was quantified using three amino sugars as biomarkers: glucosamine (GlcN), galactosamine (GalN), and muramic acid (MurA). Among these, GlcN is primarily derived from fungal cell wall residues, while MurA is used as an indicator of bacterial cell wall residues in soil. Gas chromatography–mass spectrometry was employed to determine soil amino sugars. These detected amino sugars served as representative indicators of FNC and BNC accumulation [23,24]. The specific calculation methods are as follows:

(1) $$\mathrm{FNC}=\left(179.17\times \mathrm{GlcN}\text{-}2\times \mathrm{MurA}/251.3\right)\times 9\times 179.17$$

(2) BNC=45×MurA

(3) MNC=BNC+FNC

where 179.17 is the molecular weight of GluN, 9 is the conversion factor of fungal GluN to FNC, and 45 is the conversion factor of bacterial MurA to BNC [3].

The necromass accumulation coefficient (NAC) is defined as the ratio of microbial necromass carbon to living microbial biomass carbon, representing the amount of necromass accumulated per unit of microbial biomass C. The NAC was calculated following the method described by Zhang et al. [25]. The calculation formula is as follows:

(4) $$\mathrm{NAC}=\frac{C_{\mathrm{necromass}}}{C_{\mathrm{living}\;\mathrm{microbial}}}$$

### 2.5. Metagenome Assembly

The Mag-Bind^®^ Soil DNA Kit (Omega Bio-tek, Norcross, GA, USA) was employed to extract total genomic DNA from the soil specimens, following the guidelines provided by the manufacturer. The extracted DNA was quantified using a TBS-380 fluorometer (Turner BioSystems, Sunnyvale, CA, USA), and DNA purity was evaluated with a NanoDrop 2000 spectrophotometer (Thermo Fisher Scientific, Wilmington, DE, USA). In addition, 1% agarose gel electrophoresis was employed to assess the DNA extracts’ integrity and quality.

To construct the paired-end library, the DNA extract was sheared to an average fragment length of −350 bp using a Covaris M220 ultrasonicator (Shengneng Co., Ltd., Shanghai, China), followed by library preparation with the NEXTFLEX Rapid DNA-Seq Kit (Bioo Scientific, Austin, TX, USA). Sequencing adapters containing full primer hybridization sites were ligated to the end-repaired, blunt-ended DNA fragments. Paired-end sequencing was conducted on an Illumina NovaSeq (Illumina Inc., San Diego, CA, USA) at Majorbio Bio-Pharm Technology Co., Ltd. (Shanghai, China) with a NovaSeq™ X Series 25B Reagent Kit (300 Cycle) following the manufacturer’s instructions. All sequencing data generated in this study have been submitted to the Sequence Read Archive (SRA) database of the National Center for Biotechnology Information (NCBI) following standard protocols and are publicly accessible (Accession Number: PRJNA1442876). The gene catalog was annotated using the NR database1 to acquire taxonomic and functional annotations of microorganisms, and the CAZyme database2 for the detection of particular gene families that are responsible for organic matter decomposition. The RPKM, indicating reads per kilobase per million mapped reads, served as the basis for computing the normalized abundance Figures. Table S1 contains comprehensive data regarding the genes responsible for breaking down components derived from plants and microbes.

### 2.6. Statistical Analysis

The collected data were analyzed in SPSS 22.0. One-way analysis of variance (ANOVA) was performed to evaluate the effects of fertilization treatment on all measured variables. The relationship among variables was examined using a two-tailed Pearson correlation analysis, and Origin 2021 software was utilized to analyze gene rates in the Figures. Treatment differences were assessed for statistical significance using the least significant difference (LSD) method and a one-factor ANOVA model; all statistical tests were conducted at a significance level of *p* ≤ 0.05. Soil microbial β-diversity was assessed by PCoA based on Bray–Curtis distance [26]. The circlize package (version 0.4.18) was employed to produce Circos plots [27]. The Mantel test used the ggcor R package (version 0.9.7) [28]. The random forest analysis was performed to test the key factors of CAZyme family gene abundance on the effect of MNC. The relative importance of each environmental variable was evaluated based on the %IncMSE, which was derived from the ‘randomForest’ function in R (version 4.5.3, R Core Team, Vienna, Austria). Meanwhile, the statistical significance of CAZyme family gene abundances was tested using the rfPermute function. Partial least squares path modeling (PLS-PM) was conducted using the plspm package (version 0.4.9) [29].

## 3. Results

### 3.1. Characteristics of SOC and MNC Contents Under Long-Term Application of Organic Manure and Straw

**Figure 1:**
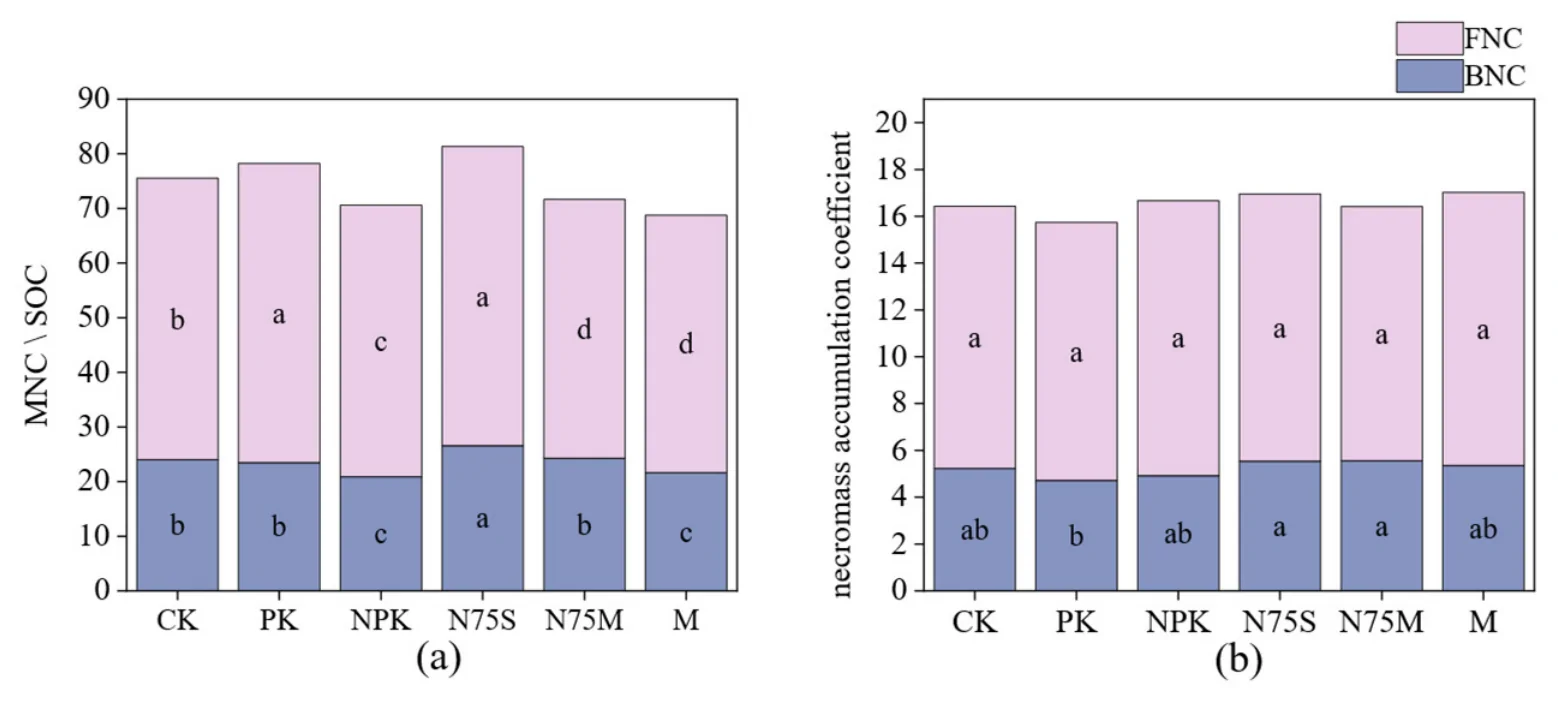
The proportions of microbial necromass carbon to soil organic carbon (**a**) and to microbial biomass carbon (**b**) under different fertilization treatments. Different lowercase letters indicate significant differences at *p* < 0.05.

The SOC, MBC and MNC in N75S, N75M and M increased by 8.6–23.1%, 7.18~12.40%, and 11.1–16.0% compared with CK, respectively (Table 1). Conversely, PK and NPK decreased by 2.88–5.85%, 4.35–10.87% and 0.37–12.79% respectively. The SOC in M and N75M significantly increased by 13.33% and 9.91% compared with N75S, respectively. Compared with CK, the MBC increased by 4.00% in PK, whereas it decreased by 14% in NPK. Compared to the CK, NPK significantly enhanced the contents of FNC, BNC, and MNC, whereas PK exhibited no significant effect. Compared with the inorganic fertilizer treatments, the N75S, N75M, and M treatments significantly elevated the contents of FNC, BNC, and MNC. Compared with N75M, N75S increased the FNC by 5.25%, resulting in a 3.25% increase in the MNC content. Meanwhile, it significantly increased the MNC/SOC (Figure 1a). The MNC/SOC in N75S and PK increased by 2.62% and 6.71% compared with CK, respectively, whereas it decreased by 7.40% in NPK. NPK, N75S, N75M, and M increased the necromass accumulation coefficient (NAC), whereas PK decreased by 4.21% (Figure 1b).

### 3.2. Soil Microbial Community Composition Under Long-Term Application of Organic Manure and Straw

**Figure 2:**
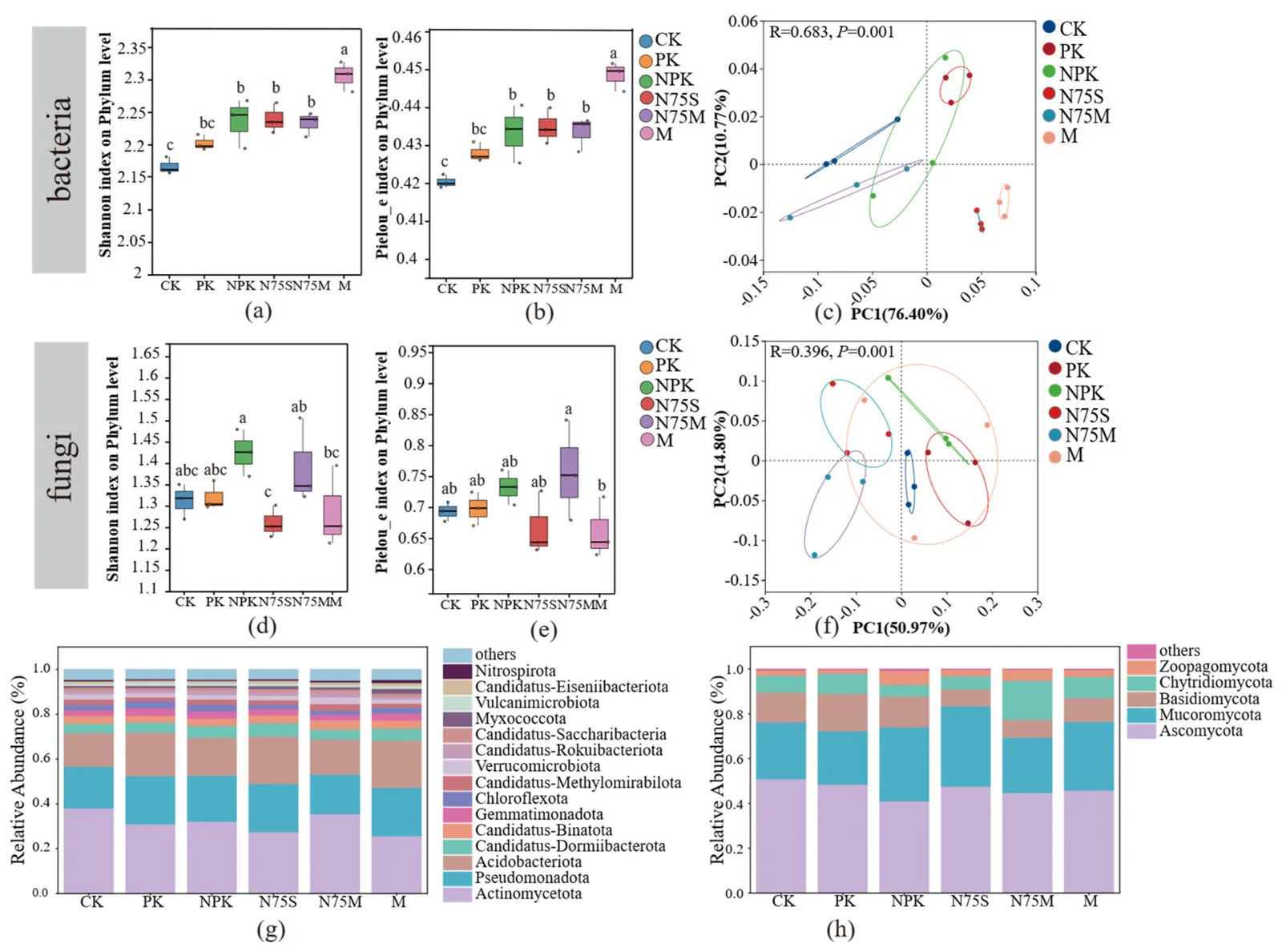
Soil microbial communities in response to different fertilization treatments. Shannon and Pielou evenness indices of bacterial (**a**,**b**) and fungal (**d**,**e**) diversity under different fertilization treatments. The boxplots display the minimum, maximum, median, first quartile, and third quartile values in the dataset. Principal component ordination analysis (PCoA) of (**c**) bacterial and (**f**) fungal communities. Relative abundances of the most important (**g**) bacterial and (**h**) fungal communities. Different lowercase letters indicate significant differences at *p* < 0.05.

Alpha diversity analyses revealed that both M and N75S reduced the fungal community’s Pielou evenness index and Shannon index, while significantly increasing those of the bacterial community (Figure 2a,b,d,e). The fungal community’s Pielou evenness index and Shannon index under N75M were 10.39% and 13.52% higher, respectively, than those under N75S, whereas the bacterial community showed no significant difference. However, bacterial and fungal diversity was higher in NPK than PK. PCoA revealed that different fertilization methods significantly affected microbial composition, explaining 68.3% and 39.6% of the bacterial and fungal community variation, respectively (Figure 2c,f). The fungal communities in PK and NPK differed from those in N75S and N75M, whereas the bacterial community in PK and M differed from that in the other treatments. Across all samples, Actinomycetota, Pseudomonadota, and Acidobacteriota were the predominant phyla, showing the highest relative abundance (Figure 2g). The abundance of Pseudomonadota increased by 18.12% and 17.09% in N75S and M compared to CK, respectively, whereas that of Actinomycetota decreased by 26.55% and 32.43%, respectively. Among the fungal community, Mucoromycota and Ascomycota were identified as the two most dominant phyla (Figure 2h). Different fertilization treatments decreased the proportion of Ascomycota.

### 3.3. Changes in CAZyme Functional Families Under Long-Term Application of Organic Manure and Straw

**Figure 3:**
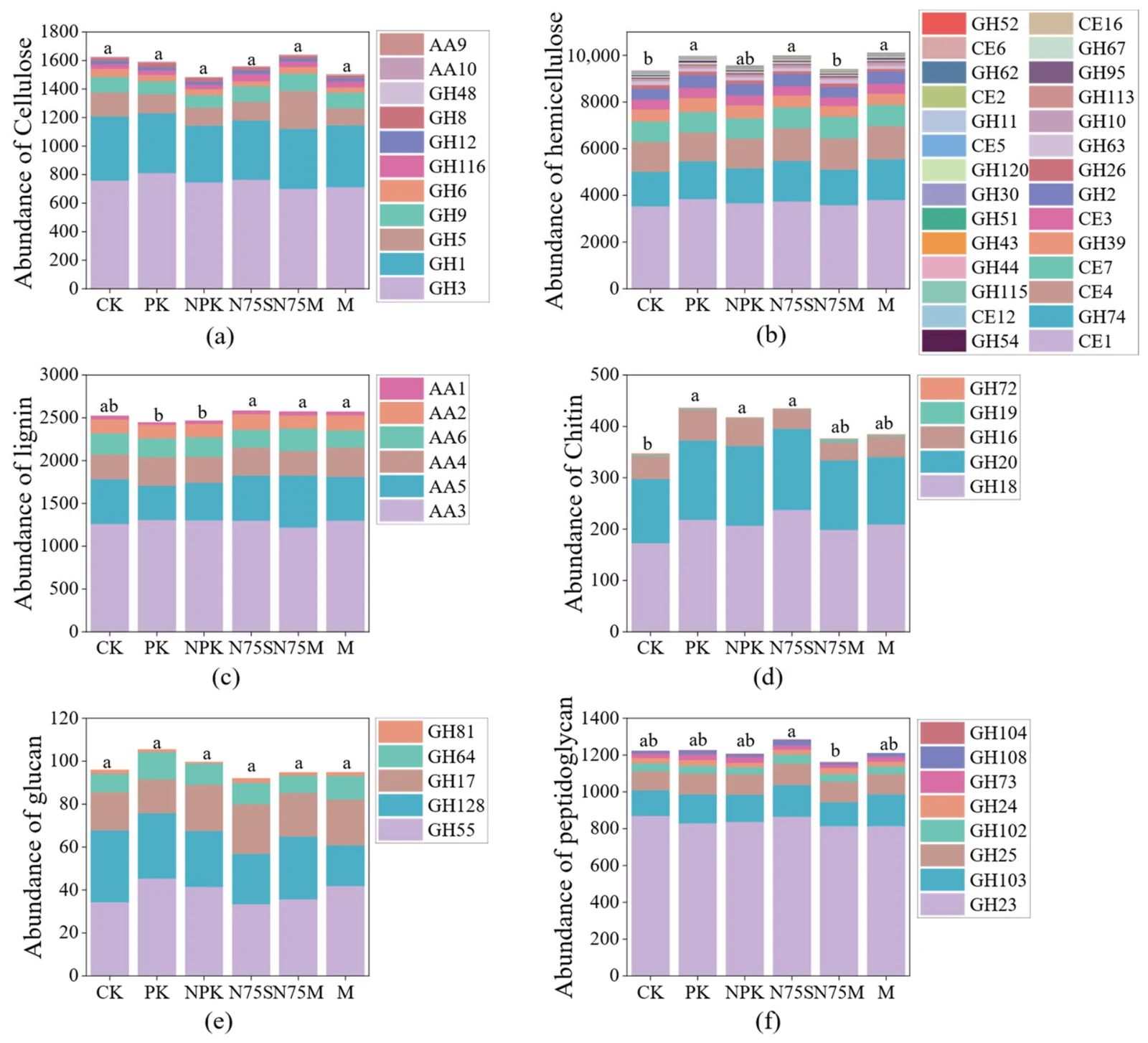
The abundance of CAZymes specifically encoding functions for the degradation of plant- and microbial-derived components under different fertilization treatments. Plant-derived: (**a**) cellulose, (**b**) hemicellulose, (**c**) lignin. Fungal-derived: (**d**) chitin, (**e**) glucans. Bacterial-derived: (**f**) peptidoglycan. Detailed information on the enzymes corresponding to each gene is provided in Table S3. Different lowercase letters indicate significant differences at *p* < 0.05.

A higher abundance of CAZymes was observed for plant-derived compound breakdown compared to microbial-derived compound decomposition. For plant-derived components, CAZyme families responsible for hemicellulose decomposition had a higher abundance than those responsible for cellulose and lignin decomposition. The abundance or composition of CAZyme family genes related to plant residue and microbial component decomposition was significantly affected by fertilization treatments (Figure 3, Table S3). In N75S, CAZymes responsible for bacterial- and fungal-derived compound decomposition declined, whereas those for plant-derived component decomposition increased compared to NPK. In detail, compared with N75M, CAZyme genes involved in plant-derived hemicellulose degradation were significantly more abundant in N75S (Figure 3b, Table S3). Among the gene families involved in plant residue degradation, CE1 and GH74 had the highest abundance, and both were significantly enhanced with increased use of organic fertilizers. The abundance of the AA4 and AA2 gene families that participate in lignin degradation was higher under N75S than under N75M and M (Figure 3c, Table S3). Compared with N75M and M, N75S showed a significantly higher abundance of the CAZyme gene families associated with degrading bacterial-derived chitin. Under N75S, the abundance of GH18 and GH20 (chitin-degrading gene families) was higher than under N75M by 23.84 and 14.55 RPKM, respectively (Figure 3d, Table S3). The abundance of CAZyme gene families implicated in peptidoglycan degradation did not exhibit a statistically significant difference between the PK and NPK treatments. Compared with CK, N75M exhibited a 56.7 RPKM decrease in the abundance of GH23, which degrades peptidoglycan (Figure 3f, Table S3).

**Figure 4:**
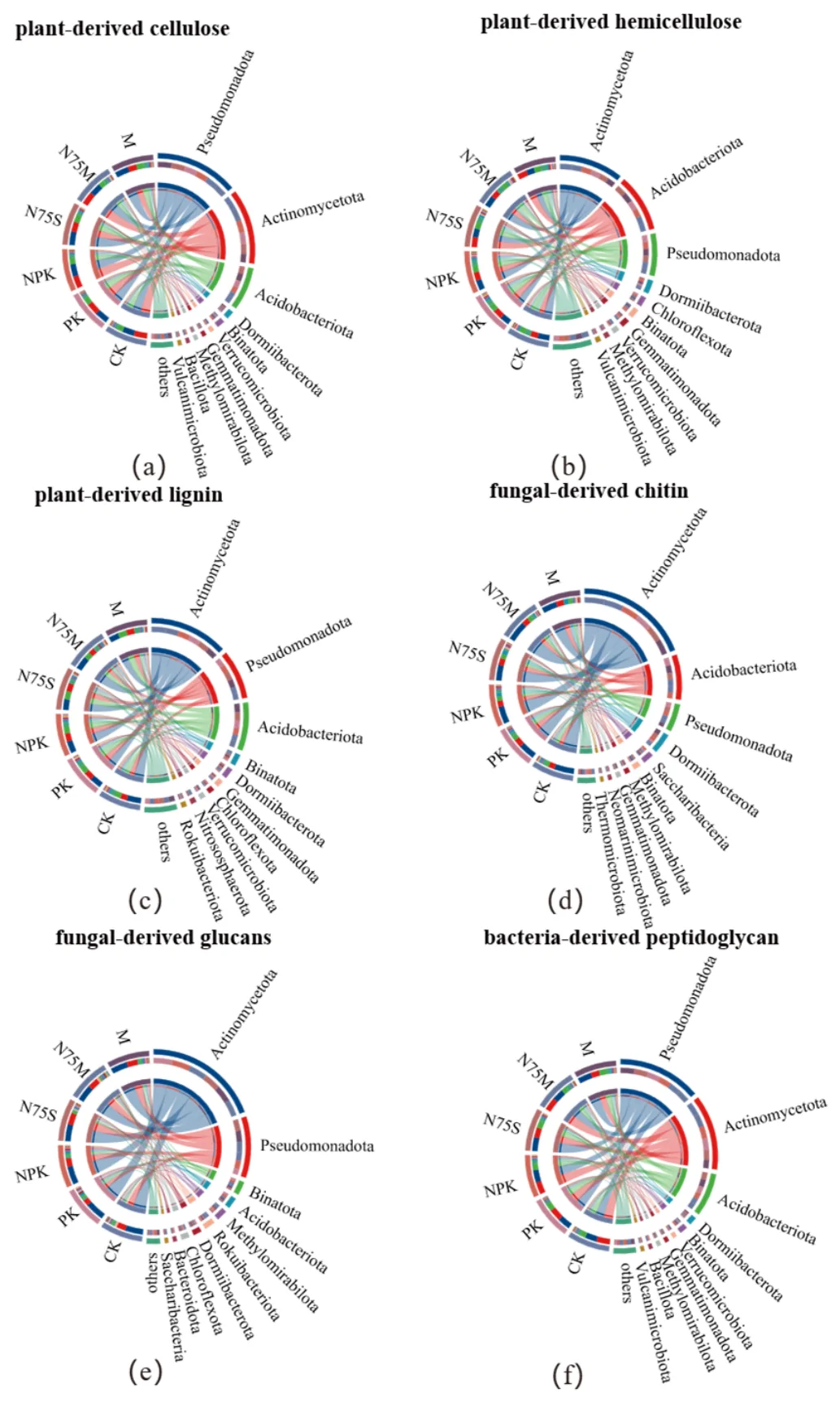
The contribution of microbial phyla encoding microbial CAZyme genes to the degradation of both plant- and microbial-derived components under different fertilization treatments. (**a**) Cellulose, (**b**) hemicellulose, and (**c**) lignin were plant-derived components, (**d**) chitin and (**e**) glucan were fungal-derived components, and (**f**) peptidoglycans were bacterial-derived components.

Bacterial communities, dominated by Acidobacteriota, Pseudomonadota, and Actinomycetota, were the primary source of CAZymes encoding MNC decomposition (Figure 4, Table S4). Compared with NPK, N75S and N75M showed an increased abundance of species associated with plant-derived cellulose and lignin degradation (Figure 4a,c, Table S4). N75S increased the abundance of species that decompose hemicellulose, chitin, and peptidoglycan, whereas that of species associated with lignin, cellulose, and glucan degradation was reduced. Indeed, Actinomycetota was the most dominant phylum in the decomposition of both plant- and fungal-derived substrates. The abundance of Actinomycetota participating in plant-derived decomposition under N75M was significantly elevated than that under M and N75S. In contrast, N75S showed a significantly higher abundance of Acidobacteriota associated with plant-derived component degradation than N75M did. Pseudomonadota primarily participated in peptidoglycan degradation. Relative to the other treatments, it showed significantly higher abundance under N75S (Figure 4, Table S4).

### 3.4. Relationships Among CAZyme Families, Microbial, and Necromass Carbon

**Figure 5:**
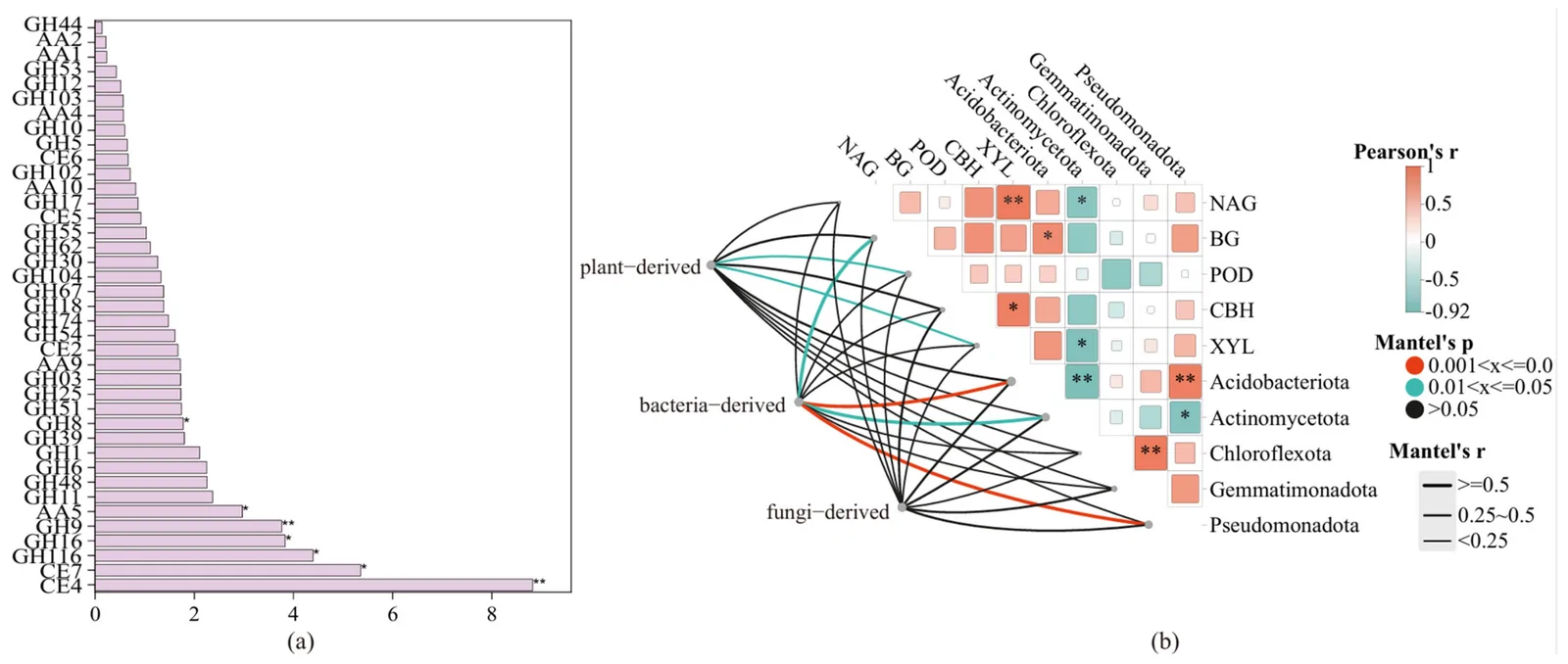
(**a**) According to the random forest model, the relative importance of predictor variables for microbial necromass carbon is expressed as the percentage increase in mean squared error (MSE%). (**b**) Relationships among specific CAZyme gene family members, enzyme activities, and microbial phyla under different fertilization treatments. * *p* < 0.05, ** *p* < 0.01.

**Figure 6:**
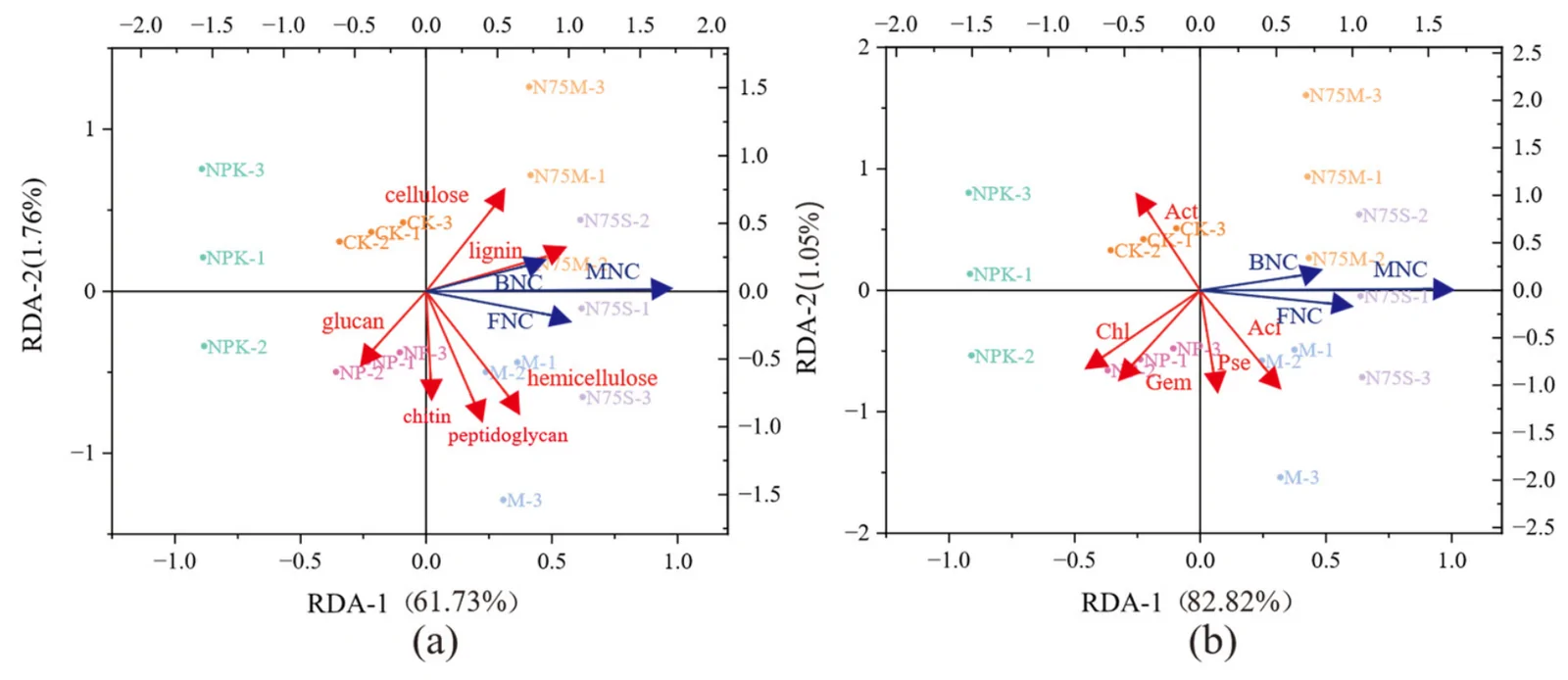
Relationship among CAZyme members with MNC, and microbial phyla with MNC across different fertilization treatments. (**a**) Relationship between MNC and the specific CAZyme gene family members. (**b**) Relationship between MNC and the microbial phyla. Act: Actinomycetota; Pse: Pseudomonadota; Aci: Acidobacteriota; Chl: Chloroflexota; Gem: Gemmatimonadetes.

According to random forest analysis, the key CAZyme gene families related to MNC were revealed (Figure 5a). The Mantel test revealed a positive relationship between gene families responsible for decomposing plant residues and POD and XYL. A significant positive correlation was observed between genes responsible for bacterial-derived degradation and Acidobacteriota, Pseudomonadota, and Actinomycetota. In addition, a significant positive correlation was observed between Acidobacteriota and BG. In contrast, Actinomycetota exhibited significant negative correlations with XYL and NAG (Figure 5b). The RDA results showed a positive association between the CAZyme gene families responsible for plant-based breakdown and the occurrence of BNC, FNC, and MNC. Degradation of glucan derived from fungi exhibited a significant negative correlation with BNC, FNC, and MNC (Figure 6a). Moreover, both fungi-derived chitin degradation genes and bacteria-derived peptidoglycan degradation genes were negatively correlated with BNC. Acidobacteriota were correlated positively with FNC, BNC, and MNC. Actinomycetota were correlated positively only with BNC, while Pseudomonadota were positively correlated only with FNC. However, Chloroflexota and Gemmatimonadota were negatively correlated with BNC, FNC, and MNC (Figure 6b). PLS analysis showed that CAZyme gene families and MNC contributions to SOC primarily mediated the effects of long-term different fertilization treatments on SOC pools (Figure 6a). BNC and FNC jointly determined the MNC content. CAZyme gene families primarily influenced MNC by modulating enzyme activities. Changes induced by fertilization methods and microbial communities accounted for the variation in CAZyme gene families (Figure 6b).

## 4. Discussion

### 4.1. Application of Organic Manure and Straw over the Long Term Promotes SOC and MNC

Twelve-year straw return and organic manure treatments can effectively enhance the SOC and MNC content, which corroborates prior findings [30,31]. In this study, M and N75M effectively enhanced the SOC content due to organic manure, which not only directly supplies exogenous organic carbon but also significantly enhances enzyme activities, thereby promoting the decomposition of organic matter as well as increasing SOC accumulation [32]. However, the PK and NPK treatments significantly decreased the SOC, as long-term application of mineral fertilizer alone provides no exogenous organic carbon, and its continuous use accelerates SOC mineralization, resulting in persistent carbon deficit [31]. In this study, the proportion of FNC in MNC exceeded that of BNC, implying that FNC is the dominant contributor to MNC formation compared with BNC [33,34]. FNC is more recalcitrant than BNC. The cell wall of bacteria is largely made up of labile compounds, for instance, peptidoglycan, while the fungal cell wall contains largely recalcitrant components, for instance, chitin. This component stability facilitates the accumulation of FNC. Soil microorganisms drive MNC accumulation through the transformation of in situ organic matter, so it is constrained by nutrient availability for BNC and FNC accumulation [35].

The PK and NPK treatments reduced the BNC content. A potential explanation for this result is that fungal biomass dominates in these systems, thereby driving bacterial exclusion or predation [36], and consequently reducing BNC. This result supports the theory that fungi decompose complex organic substrates more efficiently. Moreover, alterations in the quality of substrates may be conducive to fungal decomposition routes [37]. In NPK, the MNC/SOC decreased compared with CK. This result may be attributable to the fact that although chemical fertilizer alone increases root carbon input, it lacks exogenous labile organic carbon. Under conditions of limited total carbon availability, microorganisms preferentially utilize labile carbon and face resource competition between anabolic and catabolic metabolism. Consequently, the microbial assimilation efficiency of plant-derived carbon decreases, along with an inhibition of residue conversion into stable carbon pools [38]. A substantial proportion of plant residues do not fully undergo the MCP conversion pathway, but directly enter the soil carbon pool as partially decomposed organic matter. In the N75S treatment, FNC and MNC exhibited the highest contents and significantly higher contribution rates to SOC compared with the other treatments. However, N75M and M exhibited the highest SOC contents, but the MNC/SOC ratios were relatively lower (Figure 1a). Straw is composed largely of structurally complex organic compounds, including cellulose, hemicellulose, and lignin, and has a relatively high C/N ratio. An efficient MCP converts labile carbon into MNC, thereby achieving a high MNC/SOC [39]. Despite the fact that organic manure application significantly raised the SOC content through substantial exogenous organic carbon inputs, the residual carbon after composting is mostly stable humic substances with slow decomposition rates, resulting in a relatively lower MNC/SOC [40]. PK decreased the necromass accumulation coefficient (NAC) (Figure 1b), which may be attributable to nutrient imbalance caused by long-term phosphorus and potassium fertilization, exacerbating microbial carbon limitation. Therefore, the accelerated consumption of its own necromass to sustain metabolism led to a decrease in the NAC. N75S, N75M, and M increased the NAC; this implies a microbial carbon allocation strategy that favors biomass and necromass production over respiratory consumption, which increases MNC accumulation efficiency per unit of microbial biomass and strengthens the potential for long-term carbon storage [41].

### 4.2. Long-Term Application of Organic Manure and Straw Reshape Microbial Communities and Carbon Degradation Genes

Different fertilization treatments led to a significant divergence in microbial community structure and distinct shifts in community composition (Figure 2). Under N75S and M, there was a decrease in the abundance of oligotrophic Actinomycetota, while Acidobacteriota and Pseudomonadota increased (Figure 2g). Pseudomonadota in the eutrophic group can rapidly utilize readily decomposable cellulose and hemicellulose, leading to an increase in their abundance [42]. Acidobacteriota are proficient at degrading complex polysaccharides, and their enrichment drove the conversion of plant-derived carbon toward microbial biomass [43,44]. Under N75S and M, fungal community diversity and evenness were significantly lowered, which is consistent with previous studies that organic manure application and straw return select specific fungi as dominant species, and thus fungal diversity was reduced [45]. As simple and complex organic compounds were decomposed and utilized by microorganisms, significant alterations were observed in the community composition and diversity of soil microbes involved in organic matter decomposition, alongside the abundance of their carbon-related functional genes [46]. Across all fertilization treatments, CAZyme abundance was significantly higher for plant-derived than for microbial-derived component degradation (Figure 3, Table S3). This result was found to be consistent with previous observations [47]. Thus, fertilization increases plant-derived organic matter, prompting soil microorganisms to secrete enzymes involved in plant carbon decomposition (Table S1), resulting in the enrichment of genes related to plant-derived carbon degradation (Table S2), thereby increasing genes associated with the degradation of carbon derived from plants [48]. The abundance of carbon-degrading genes in plants such as cellulose, hemicellulose, and lignin did not change synchronously; fertilization resulted in a decrease in the abundance of cellulose-degrading genes, whereas those associated with hemicellulose and lignin degradation increased. This is related to the organic substrate degradation capacity and preference [49]. In the microbiome, there were significantly more bacterial-derived CAZyme genes involved in component degradation than fungal-derived ones (Figure 2). Following different fertilization treatments, most microorganisms preferentially utilize labile substrates to obtain nutrients, carbon, and energy under nutrient-rich conditions. Consequently, enhanced expression is observed in genes participating in labile carbon degradation [50].

In this study, the total abundance of CAZyme genes was substantially elevated under N75S, indicating enhanced microbial functional activity or potential [51]. N75S, N75M, and M increased the abundance of encoded hemicellulose and lignin degradation, whereas PK and NPK decreased them (Figure 3b; Table S3). This may be attributable to the fact that energy substrate availability is a key determinant of the production of carbon-degrading enzymes [52]. Through providing energy sources and additional substrates for soil enzymes responsible for plant-derived component decomposition, organic manure and straw return treatments facilitate both gene expression and enzyme synthesis in these enzymes [53]. N75S upregulated CAZyme genes involved in bacterial component degradation, whereas N75M decreased it (Figure 3f; Table S3). Organic manure provides ample labile organic carbon, allowing microorganisms to acquire nutrients without synthesizing large amounts of bacterial cell wall-degrading enzymes [54]. In N75S, there was a notably greater presence of CAZyme gene families engaged in degrading fungal-derived chitin compared to N75M and M (Figure 3d; Table S3). Under straw return conditions, the exogenous input of complex organic carbon stimulates the growth of specific fungal communities, leading to increased chitin content, which in turn enhances the abundance of CAZyme genes responsible for fungal-derived chitin [55]. In contrast, organic manure has undergone partial decomposition and is rich in readily decomposable small-molecular-weight organic matter. This type of organic matter is primarily decomposed by bacteria. Therefore, the demand for organic matter decomposition by fungi is substantially reduced. This results in lower fungal community abundance in the organic manure treatments. Meanwhile, the abundance of CAZyme genes associated with fungal-derived chitin degradation was also lower than that under N75S. Among the microbial communities harboring CAZyme genes, bacteria are the predominant group (Figure 4, Table S4). A prior investigation that examined bacterial and fungal community structure across multiple spatial scales reported that bacterial functional genes contributed 1.2 times more than fungal counterparts to recalcitrant carbon mineralization [56]. Among the different fertilization treatments, Actinomycetota was the predominant phylum that harbored the CAZyme family genes responsible for degrading plant- and fungal-derived components, which is consistent with a past study on soils under different vegetation types [57]. Pseudomonadota promoted carbon degradation under N75S [58]. The CAZyme gene pool for bacterial component degradation was notably contributed by Pseudomonadota, thereby further evidence for the role of Pseudomonadota in soil carbon degradation was provided by this result. Therefore, plant residues and bacterial necromass exhibited greater carbon degradation potential under N75S. To summarize, by enhancing metabolic activity, increasing CAZyme gene abundance, and elevating extracellular enzyme activity, microorganisms promoted the degradation of dead biomass under different fertilization treatments, thereby further enhancing the SOC pool content.

### 4.3. Mechanistic Pathways of Long-Term Straw and Organic Manure Application in Regulating Soil Microbial Necromass Carbon Accumulation

**Figure 7:**
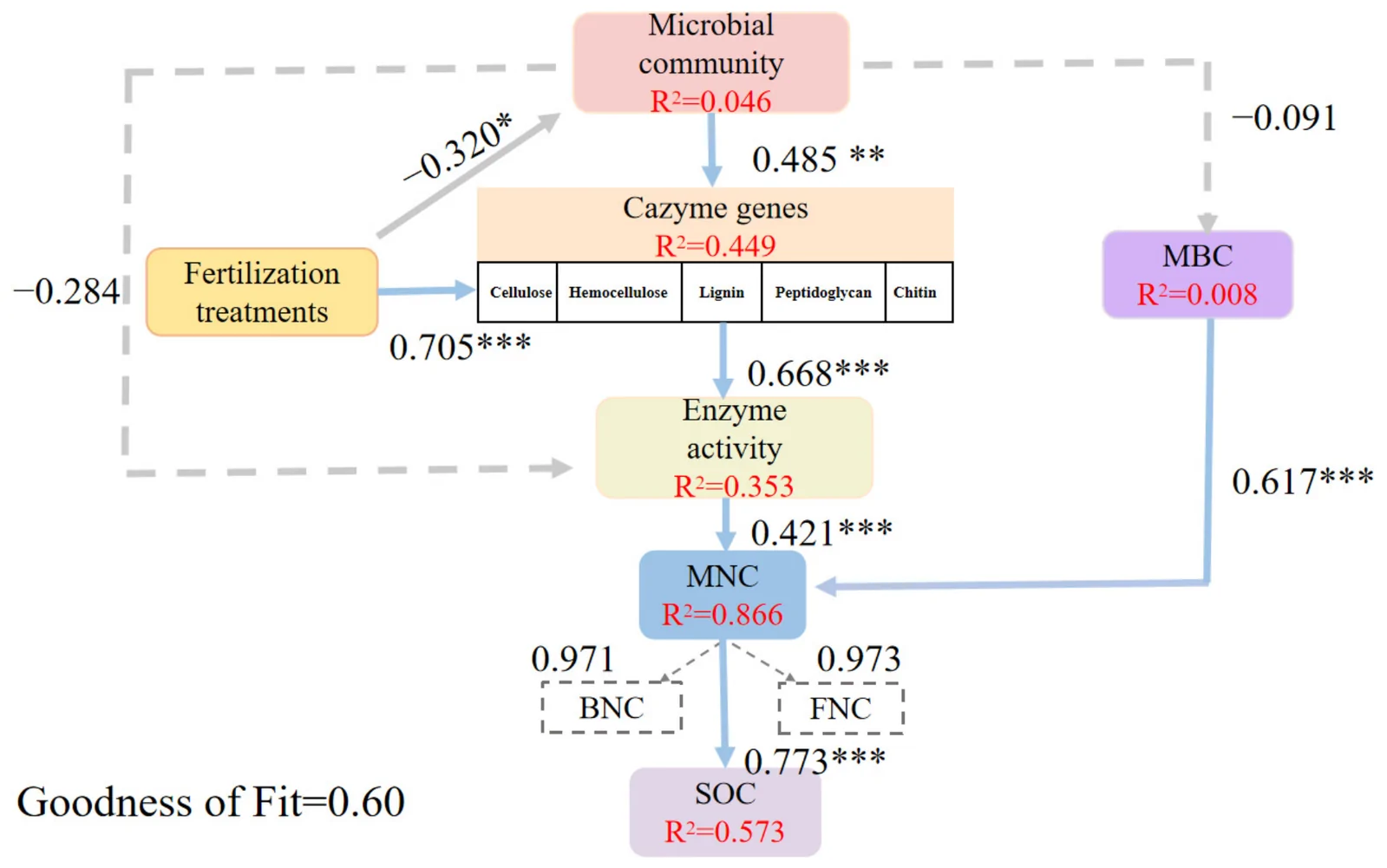
Direct and indirect factors affecting microbial necromass carbon accumulation under different fertilization treatments. Significant positive correlations are represented by solid blue lines, whereas non-significant correlations are represented by dashed gray lines. Values on the arrows are standardized path coefficients. Arrow width is proportional to the magnitude of the path coefficients. * *p* < 0.05, ** *p* < 0.01, *** *p* < 0.001.

Numerous studies have demonstrated that functional genes modulate extracellular enzyme activity [59]. Extracellular enzyme activities were found to be significantly correlated with the abundance of genes involved in carbon degradation, suggesting that CAZyme genes may regulate SOC formation. Soil extracellular enzyme activity and microorganisms have a direct relationship, which further accounts for the significant regulatory function of microbes during the dynamic formation of MNC (Figure 5). In this study, changes in carbon-degrading genes will ultimately influence SOC accumulation by altering the MNC (Figure 7). Fertilization treatments affected the supply of plant-derived organic matter to the soil [60]. The organic matter is mainly composed of lignin and cellulose [61], which drives microorganisms to encode an increased number of functional genes that have the specificity to degrade plant-derived lignin and cellulose [62]. Through the in vivo turnover pathway, small-molecule substrates that are readily decomposable and derived from plant necromass are subsequently converted by microorganisms into microbial biomass [63]. MNC was positively correlated with genes that participate in plant-derived hemicellulose and lignin degradation. However, a significant positive correlation was found between the degradation of bacterial-derived peptidoglycan and FNC, rather than BNC. This indicates that the high expression of hemicellulose- and lignin-degrading genes acts directly on plant residues, providing abundant available substrate for bacteria, promoting rapid bacterial proliferation and death. However, BNC is merely a rapidly turned-over intermediate product, whose overall accumulation is limited by substrate supply and intense re-decomposition processes; therefore, it showed no significant positive correlation with the peptidoglycan-degrading genes. However, the nutrients released from decomposed bacterial necromass are primarily utilized by fungi. Ultimately, through fungal metabolism, into more recalcitrant and mineral-protected chitin and glucan, thus accumulate steadily as FNC [64].

N75S is rich in complex structural compounds such as lignin and hemicellulose, and enhances the abundance of eutrophic bacteria in the Acidobacteriota and Pseudomonadota phyla (Figure 2). There was a notable rise in the abundance of genes responsible for producing enzymes that degrade hemicellulose and lignin from plants. This process drives the MCP: microorganisms rapidly proliferate by utilizing readily degradable substrates and convert a portion of plant-derived carbon into microbial biomass, providing energy for subsequent MNC formation [47]. Meanwhile, the abundance of chitin and peptidoglycan degradation genes increased, indicating rapid microbial community turnover under high exogenous carbon input conditions. This process promotes the decomposition of cell wall components, driving more easily mineralizable labile carbon sources through the microbial assimilation pathway into the stable necromass carbon pool. This reflects the core mechanism of the MCP: converting easily mineralized carbon sources into stable necromass carbon pools [65]. The results of this study also indicated that, in the organic manure treatment, there was a rise in the abundance of eutrophic bacteria such as Pseudomonadota, while CAZyme genes that degrade cellulose, hemicellulose, and lignin were upregulated. This led to increased activities of POD, CBH, and XYL (Table S2), enabling microorganisms to preferentially utilize fresh plant-derived carbon for rapid metabolism. At the same time, with an abundant supply of substrate, the microbial demand for the degradation of existing microbial necromass is reduced [66]. Peptidoglycan- and glucan-degrading genes were suppressed. Consequently, the decomposition rates of fungal and bacterial residues declined, allowing partial preservation of MNC. However, the proportion of microbial-derived carbon is diluted by the considerable input of plant-derived carbon. Concurrently, microbial metabolic turnover is relatively rapid. Although necromass production and decomposition remain in a dynamic equilibrium, the net accumulation efficiency was limited [67], resulting in a reduction in the MNC/SOC. The mechanism of MNC accumulation under organic manure application differs from that under straw return. Organic manure tends to promote rapid nutrient turnover, whereas straw return is more conducive to building a persistent and stable SOC pool. The correlations of both CAZyme functional genes and microbial community abundance reveal the regulatory function of soil microorganisms in carbon cycling, as well as the essential role of CAZyme families in the turnover of carbon sources. Variations in CAZyme genes responsible for encoding microbial necromass carbon may influence microbial metabolism through the MCP, facilitating the formation and accumulation of SOC.

## 5. Conclusions

This investigation clarifies both the mechanisms through which long-term organic amendment enhances SOC accumulation, and the routes of microbial-derived carbon mediated by variations in CAZyme genes. The results of the 12-year field experiment demonstrated that long-term straw return markedly enhanced SOC accumulation. Meanwhile, the abundances of hemicellulose- and lignin-degrading genes, and the abundance of Pseudomonadota were all significantly increased. This further enhanced enzyme activities and accelerated the transformation of plant residues into MNC, thereby significantly promoting the accumulation of both BNC and FNC. Meanwhile, the increases in chitin- and peptidoglycan-degrading genes enhanced the turnover efficiency of the MCP, thereby increasing both the MNC and the contribution of MNC to SOC. Organic fertilizer treatments significantly enhanced SOC accumulation. It increased CAZyme gene abundance for plant-derived carbon degradation, driving microorganisms to preferentially metabolize fresh plant-derived carbon. When substrates are abundant, microorganisms reduce the decomposition of microbial remnants, leading to a decline in peptidoglycan- and glucan-degrading genes, while MNC increased significantly. However, plant-derived carbon input diluted the microbial carbon proportion. Coupled with rapid microbial turnover and limited necromass accumulation efficiency, this ultimately led to a decline in the MNC/SOC. Overall, through clarification of these microscale processes, our findings not only supported the underlying connection between MNC and microbial CAZyme genes but also advanced insights into the potential for soil carbon storage across larger regions. This study provides valuable insights into how the SOC pool and microbial CAZyme genes are connected underlyingly under long-term fertilization. However, it should be noted that the relationship between changes in gene abundance and net MNC accrual is not simply linear. Therefore, the CAZyme gene abundance revealed by metagenomics should be interpreted as a “functional indicator” of microbial carbon degradation potential, rather than a direct quantitative predictor of net MNC accumulation. Future studies are needed to further elucidate the conversion efficiency and key regulatory nodes from specific gene expression to net necromass stabilization.

## Figures and Tables

**Table 1:** Soil organic carbon and microbial necromass carbon under different fertilization treatments.

	SOC (g/kg)	MBC (g/kg)	FNC (g/kg)	BNC (g/kg)	MNC (g/kg)
CK	10.77 ± 0.1 c	0.5 ± 0.01 b	5.6 ± 0.04 c	2.61 ± 0.01 bc	8.21 ± 0.02 c
PK	10.46 ± 0.59 c	0.52 ± 0.01 bc	5.73 ± 0.11 c	2.45 ± 0.11 c	8.18 ± 0.07 c
NPK	10.14 ± 0.17 c	0.43 ± 0.02 c	5.05 ± 0.16 d	2.11 ± 0.04 d	7.16 ± 0.14 d
N75S	11.7 ± 0.2 b	0.56 ± 0.01 a	6.41 ± 0.09 a	3.11 ± 0.01 a	9.52 ± 0.19 a
N75M	12.86 ± 0.48 a	0.56 ± 0.02 a	6.09 ± 0.12 b	3.12 ± 0.0 6a	9.22 ± 0.09 b
M	13.26 ± 0.04 a	0.54 ± 0.02 ab	6.25 ± 0.05 ab	2.87 ± 0.02 b	9.12 ± 0.12 b

Note: Different lowercase letters indicate significant differences at *p* < 0.05. SOC: soil organic carbon; MBC: microbial biomass carbon; FNC: fungal necromass carbon; BNC: bacterial necromass carbon; MNC: microbial necromass carbon.

## Data Availability

The original data presented in the study are openly available in the NCBI database at BioProject PRJNA1442876.

## Supplementary Materials

The following supporting information can be downloaded at: https://www.mdpi.com/article/10.3390/microorganisms14091970/s1, Table S1. Functional classification of glycosyl hydrolases and auxiliary activities enzymes involved in the degradation of plant and microbial compounds; Table S2. The enzymes activity under different fertilization treatments; Table S3. Abundance of microbial enzyme genes for decomposition of plant- and microbial-derived components under different fertilization treatments. Different lowercase letters indicate a significant differences among different fertilization treatments (*p* < 0.05); Table S4. Abundance of microbial phyla under different fertilization treatments. Different lowercase letters indicate a significant differences among different fertilization treatments (*p* < 0.05).

## Abbreviations

The following abbreviations are used in this manuscript:

MNC	Microbial Necromass Carbon
BNC	Bacterial Necromass Carbon
FNC	Fungal Necromass Carbon
MCP	Microbial Carbon Pump
AA	Auxiliary Activity
GH	Glycoside Hydrolases
SRA	Sequence Read Archive
NAC	Necromass Accumulation Coefficient
LSD	Least Significant Difference
PLS-PM	Partial Least Squares Path Modeling
