# Supplementary material for: Long-Term Organic Fertilization Drives Soil Organic Carbon Accumulation in Black Soil Through Microbial Necromass Carbon and CAZyme-Mediated Carbon Turnover
Source: Microorganisms. 2026 Sep 7;14(9):1970. doi: 10.3390/microorganisms14091970 (PMC13609135; doi:10.3390/microorganisms14091970)
Supplement: Supplementary file 1 [file microorganisms-14-01970-s001.zip › microorganisms-4520534-supplementary.pdf]

Long-term organic fertilization explains soil organic carbon accumulation in black soil through microbial necromass carbon and CAZyme-mediated carbon turnover

Table S1. Functional classification of glycosyl hydrolases and auxiliary activities enzymes involved in the degradation of plant and microbial compounds.

Components	CAZymefamilies	Description
Cellulose-derived	AA10<br>AA9	lytic polysaccharide monooxygenase
	GH1	β-glucosidase,（EC 3.2.1.21）
	GH116<br>GH3	β-glucosidase
	GH12<br>GH9	endoglucanase
	GH48	reducing end-acting cellobiohydrolase
	GH5	β-glucosidase;<br>endoglucanase
	GH6	cellobiohydrolase
Hemicellulose-derived	GH8	endoxylanase
	CE1<br>CE12<br>CE16<br>CE2<br>CE3<br>CE4<br>CE5<br>CE6<br>CE7	acetyl xylan esterase,（EC 3.1.1.72）
	GH10<br>GH11	endoxylanase
	GH113<br>GH26	β-mannanase (EC 3.2.1.78)
	GH120	β-xylosidase
	GH2	β-galactosidase;<br>β-glucuronidase
	GH115	xylan α-1,2-glucuronidase
	GH30	endo-β-1,4-xylanase (EC 3.2.1.8)
	GH39	β-xylosidase;<br>α-L-arabinofuranosidase
	GH43	β-xylosidase;<br>endoxylanase
	GH44	Xyloglucanase;<br>endoglucanase
	GH51	α-L_x0002_arabinofuranosidase
	GH52	β-xylosidase
	GH54<br>GH62	α-L-arabinofuranosidase
	GH63	a-glucosidase (EC 3.2.1.20)
	GH67	α-glucuronidase, EC 3.2.1.139
	GH74	xyloglucanase (EC 3.2.1.151)
	GH95	α-L-fucosidase, EC 3.2.1.51
Lignin-derived	AA1	Laccase;<br>p-diphenol:oxygen;<br>oxidoreductase/ferroxidase, (EC 1.10.3.2)
	AA2	manganese peroxidase, (EC 1.11.1.13);<br>lignin peroxidase, (EC 1.11.1.14);<br>peroxidase, EC 1.11.1.-)
	AA3	glucose 1-oxidase, (EC 1.1.3.4)
	AA4<br>AA5	Oxidase, (EC 1.1.3.-)
	AA6	1,4-benzoquinone reductase, (EC. 1.6.5.6)
Glucans-derived	GH128<br>GH17<br>GH64	endo-1,3-β-glucanase

Components	CAZymefamilies	Description
Chitin-derived	GH81	
	GH55	exo-β-1,3-glucanase; endo-1,3-β-glucanase
	GH20	N-acetylβ-glucosaminidase
	GH16	endo-xyloglucanase (EC 3.2.1.151)
	GH18	chitinase (EC 3.2.1.14)
	GH19	
	GH72	β-1,3-glucanosyltransglycosylase
Peptidoglycan-derived	GH102	peptidoglycan lytic transglycosylase
	GH103	
	GH104	
	GH23	Lysozyme; peptidoglycan lytic transglycosylase
	GH108	lysozyme
	GH24	
	GH25	
	GH73	peptidoglycan hydrolase with endo-β-N-acetylglucosaminidase specificity

Table S2. The enzymes activity under different fertilization treatments.

	NAG	BG	POD	CBH	XYL
CK	17.71±3.5c	554.12±15.07e	1805.27±53.51bc	54.63±5.83e	174.55±3.99d
PK	27.21±5.83bc	601.97±5.84d	1756.63±30.58c	83.13±1.26d	190.66±2.72c
NPK	31.52±3.32b	640.47±12.3c	1648.53±45.86d	108.48±3.9c	190.11±0.74c
N75S	32.28±5.11b	895.6±5.23a	1932.29±34.4a	139.83±4.89a	201.24±4.83bc
N75M	32.79±5.11b	588.92±26.15d	1867.43±19.11ab	115.91±1.45bc	196.01±4.28c
M	50.28±3.32a	745.96±8.92b	1826.89±7.64bc	130.83±4.54ab	210.96±6.3ab

Note: Different lowercase letters indicate a significant difference in different fertilization treatments ($P < 0.05$).

NAG: α-glucosidase; BG: β-glucosidase; POD: peroxidase; CBH: cellulase; XYL: β-xylosidase.

Table S3. Abundance of microbial enzyme genes for decomposition of plant- and microbial-derived components under different fertilization treatments. Different lowercase letters indicate a significant differences among different fertilization treatments ($P < 0.05$).

Components	CAZyme families	CK	PK	NPK	N75S	N75M	M
Cellulose-derived	GH3	758.9± 5.2abc	812.36±23.21a	747.55±26.7bc	764.56±15.5ab	701.51±9.8c	713.23±23.6bc
	GH1	450.17±11.67a	417.51±9.54ab	397.42±21.03b	415.85±12.78ab	420.75±11.92ab	432.35±18.1ab
	GH5	169.53±13.27b	135.92±6.05b	125.38±2.89b	130.42±9.24b	266.51±48.45a	121.45±4.5b
	GH9	107.17±2.03a	89.43±4.54b	87.6±5.45b	109.84±1.12a	119.84±9.95a	109.01±3.27a
	GH116	34.19±5.11bc	31.74±2.09c	28.88±2.82c	51.12±2.71a	37.41±2.55bc	41.74±3.57ab
	GH6	55.85±3.22a	45.15±2.44b	43.51±1.93bc	34.63±2.92d	47.33±2.39b	37.35±1.29cd
	GH8	20.82±4.52b	30.9±0.93a	26.05±3.19ab	23.07±1.27b	19.04±1.21b	19.57±1.49b
	GH12	23.66±1.13a	23.14±2.99a	24.09±3.2a	26.5±3.06a	22.8±2.64a	23.85±4.05a
	GH48	2.61±1.08a	3.32±0.16a	1.74±1.24a	2.13±0.3a	2.03±0.45a	3.73±a
	AA9	0.56±0.31ab	0.15±0.15ab	0.15±0.15ab	0.52±0.02ab	0.9±0.48a	0±0b
	AA10	1.13±0.24a	0.67±0.2ab	0.19±0.1b	0.23±0.11b	0.95±0.14ab	0.55±0.23ab
Hemicellulose-derived	CE1	3532.88±29.8d	3842.92±19.18a	3671.23±84.07bc	3756.64±14.42ab	3590.86±29.39cd	3745.43±30.59a
	GH74	1481.77±59.88bc	1615.52±16.22b	1491.29±21.85bc	1747.61±16.7a	1516.01±14.48bc	1748.79±39.03a
	CE4	1288.6±26.92bc	1250.5±5.05c	1279.29±14.58c	1396.55±1.11a	1338.08±4.74b	1458.78±28.19a
	CE7	867.05±13.64b	864.81±10.3b	863.85±1.02b	886.85±13.22ab	932.38±23.96a	880.96±10.28ab
	GH39	521.01±28.05bc	612.34±5.58a	559.97±38.85ab	507.22±14.32bc	451.19±8.57c	497.18±33.31bc
	CE3	411.34±12.64ab	434.55±8.19a	424.22±16.18ab	398.92±6.45bc	370.97±1.9c	431.92±4.94a
	GH2	473.86±28.91bc	541.83±9.03a	486.14±28.97abc	529.6±10.11ab	448.89±3.86c	498.36±9.05ab
	GH26	157.35±7.57a	154.21±5.41a	155.43±9.61a	116.2±5.67b	153.9±7.14a	111.96±2.42b
	GH63	159.66±6.17a	121.62±3.59c	128.08±2.52bc	142.93±7.78bc	163.71±13.03a	142.42±4.27bc
	GH10	72.23±9.42ab	90.94±1.28ab	90.82±8.71ab	92.49±4.28a	69.25±9.91b	101.3±4.83a
	GH113	43±3.66c	49.34±3.26bc	48.76±5.24bc	46.43±4.11bc	37.35±3.43c	66.66±2.37a
	GH95	44.33±3.67a	41.26±2.98a	43.81±6.99a	46.41±2.88a	49±2.62a	48.88±6.45a
	GH67	35.95±4.83bc	48.56±3.74a	45.58±5.63ab	48.49±2.59a	31.33±4.29c	57.43±3.02a
	CE16	34.64±2.38ab	39.93±5.47a	38.51±5.5a	33.44±4.09ab	29.89±2.71ab	28.19±1.84b
	GH54	30.69±1.44c	54.43±2.47a	34.84±3.71bc	32.81±1.67bc	26.88±2.26c	40.23±2.79b
	CE12	29.97±2.13bcd	38.29±2.02a	24.43±2.46d	27.16±1.59cd	31.48±1.59bc	28.64±0.89bcd
	GH115	26.2±4.69a	32±1.4a	27.56±2.51a	29.34±1.74a	23.04±1.95a	34.33±3.38a
	GH44	20.78±4.38a	21.76±0.81a	20.45±2.7a	34.1±7.33a	28.01±1.92a	27.2±2.83a

Components	CAZyme families	CK	PK	NPK	N75S	N75M	M
	GH43	16.27±1.4e	20.06±0.34de	25.64±0.87abc	28.62±0.51a	21.92±2.54cd	36.07±1.05ab
	GH51	15.32±1.99b	15.81±0.08b	13.41±0.65b	19.87±2.52a	16.47±2.08b	11.79±1.26b
	GH30	16.72±1.47b	16.05±2.9b	15.1±1.37b	23.73±1.04a	22.41±2.65ab	30.02±3.74ab
	GH120	13.25±1.36a	11.06±0.62a	12±2.62a	12.85±1.05a	13.46±1.21a	15.29±0.73a
	CE5	11.45±1.86abc	15.48±0.52a	13.88±5.01ab	5.91±1.09c	9.37±0.72abc	5.13±0.95bc
	GH11	8±0.92bc	15.31±2.05a	11.04±3.52ab	6.67±0.62bc	4.56±1.16c	7.97±0.58bc
	CE2	6.61±0.58c	6.57±1.41c	6.76±1.01c	9.93±1.52ab	8.23±1.52bc	12.2±0.41a
	GH62	6.07±2.2a	6.3±1.42a	8.37±3.07a	10.91±1.2a	5.83±0.66a	13.2±1.84a
	CE6	2.39±0.82c	2.57±0.18bc	3.54±1.14abc	5.37±0.56abc	4.41±1.19abc	6.7±0.78ab
	GH52	1.38±0.64ab	0.59±0.3ab	0.21±0.1b	0.12±0.08b	2.44±1.32a	0.4±0.39ab
Lignin-derived	AA3	1264.19±31.84ab	1308.02±12.3a	1304.92±28.93a	1298.25±7.43a	1220.53±28.34b	1285.06±8.18a
	AA5	519.16±27.89b	399.29±13.78c	437.02±24.11c	538.48±13.5b	608.54±27.75a	485.15±14.07b
	AA4	291.02±5.09c	334.95±4.91a	304.41±4.23bc	326.62±13.11ab	283.2±11.73c	352.85±9.93a
	AA6	246.86±7.47ab	217.79±2.23bc	229.78±1.89bc	204.75±9.39c	263.91±18.76a	202.81±12.85c
	AA2	163.13±12.36ab	158.46±8.33ab	154.57±6.1b	181.73±7.8a	153.1±3.69b	174.35±8.73ab
	AA1	38.06±3.59a	27.16±0.69b	35.08±2.42ab	36.47±2.2a	42.95±5.24a	41.56±3.33ab
Glucans-derived	GH55	34.48±1.53b	45.35±0.95a	41.59±5.51ab	32.4±2.41b	35.76±1.23b	36.83±3.25ab
	GH128	33.38±4.06a	30.47±1.78ab	26.03±3abc	24.3±2.17bc	29.26±3.11ab	20.73±1.58c
	GH17	17.79±0.36ab	15.78±2.22b	21.47±1.98ab	20.67±2.57a	20.33±1.51ab	18.91±1.44ab
	GH64	8.53±2.24ab	12.77±0.97a	9.81±1.69a	11.02±1.11a	8±1.12ab	13.96±1.61a
	GH81	1.75±0.81a	1.14±0.27a	0.65±0.28a	2.56±0.56a	1.42±0.33a	1.34±0.18a
Chitin-derived	GH18	173.1±7.65d	218.34±5.67ab	206.87±7.03bc	222.75±18.32a	198.91±5.7bcd	203.99±2.99abc
	GH20	124.34±8.74c	154.36±4.56a	155.54±5.49a	149.98±7.65a	135.43±2.62bc	123.08±4.05bc
	GH16	44.44±3.76bc	59.76±3.94a	52.34±6.55ab	38.11±1.85c	34.52±2.36c	45.79±3.48c
	GH19	3.15±1.45ab	2.46±1.2ab	1.25±0.58b	1.77±0.81b	6.06±1.28a	0.42±1.52ab
	GH72	2.08±0.19ab	0.9±0.49b	1.31±0.7ab	0.95±0.24ab	0.72±0.4b	0.96±0.59ab
Peptidoglycan-derived	GH23	871.07±10.4a	829.36±5.04bc	837.59±14.18abc	871.21±10.27ab	814.36±7.84c	823.72±15.02c
	GH103	138.26±5.49cd	157.03±2.15abc	147.9±3.74cd	174.44±5.37ab	129.53±14.6d	175.78±2.86a
	GH25	103.99±4.96b	114.06±1.33ab	112.16±1.65ab	120.21±1.94a	111.71±4.74ab	104.29±2.91ab

Components	CAZyme families	CK	PK	NPK	N75S	N75M	M
	GH102	42.45±3.82ab	41.17±1.16ab	36.12±3.26b	53.05±5.51a	41.74±2.06ab	34.21±3.09ab
	GH24	29.32±11.4a	32.31±2.65a	25.65±2.99a	26.77±1.78a	33.74±1.9a	26.22±1.25a
	GH73	24.15±2.19ab	30.79±3.74a	31.14±4.22a	24.12±3.12ab	17.42±4.7b	33.62±3.42ab
	GH108	13.58±1.45bc	20.79±2.59bc	15.46±2.31b	26.46±1.53a	11.51±1.94c	24.47±3.5bc
	GH104	0.24±0.24a	1.07±0.81a	0.17±0.17a	1.64±0.79a	0.87±0.87a	0.64±0.21a

Table S4. Abundance of microbial phyla under different fertilization treatments. Different lowercase letters indicate a significant differences among different fertilization treatments ($P < 0.05$).

Components		CK	PK	NPK	N75S	N75M	M
Cellulose-derived	Actinomycetota	571.52±25.18a	448.03±14.15bc	441.72±11.64bcd	402.92±19.05cde	365.08±22.61e	500.95±38.72b
	Acidobacteriota	276.25±22.98c	370±7.32a	268.49±19.71c	350.34±9.5a	334.64±5abc	271.71±14.19c
	Pseudomonadota	195.49±8.29ab	231.94±5.68ab	222.96±22.82ab	228.29±3.9ab	220.82±8.61ab	193.74±11.58b
	Chloroflexota	109.96±7.69ab	133.47±13.09ab	126.65±19.82ab	89.86±0.98b	130.12±12.92ab	84.47±5.61b
	Dormiibacterota	75.75±4.77c	77.75±2.21c	93.78±4.67bc	124.24±10.52a	80.08±3.15bc	82.08±7.58bc
	Nitrososphaerota	74.33±14.06b	15.1±3.6b	21.14±4.25b	19.01±3.33b	45.12±4.32b	175.5±48.45a
	Methylomirabilota	51.8±1.71ab	44.57±0.73b	50.26±1.94ab	51.32±1.61ab	53.31±1.74a	51.65±2.62ab
	Binatota	51.56±2.86ab	43.98±3.33bc	39.28±3.56b	48.69±2.15bc	46.35±2.9bc	60.83±2.59a
	Gemmatimonadota	32.95±1.52c	41±3.67abc	42.41±3.44abc	38.79±1.72abc	45.14±5.07ab	34.75±3.47bc
	Vulcanimicrobiota	18.55±1.64d	35.71±1.39a	27.18±1.53bc	29.08±3.16abc	25.32±3.2cd	18.81±1.49d
	others	157.58±10.86bc	141.77±1.71c	142.99±1.66c	169.16±5.27b	198.14±13.78a	160.49±4.13bc
Hemicellulose-derived	Actinomycetota	2702.81±90.9a	2380.01±41.14b	2398.71±68.61b	2134.8±8.95c	2037.04±62.54c	2499.54±111.61ab
	Acidobacteriota	1765.23±171.57c	2294.75±27.71ab	1962.66±160.8bc	2519.06±66.88a	2351.67±69.86a	1799.45±156.31c
	Pseudomonadota	1397.11±65.07bc	1743.61±35.78a	1624.6±89.83a	1654.14±33.9a	1542.9±38.09ab	1294.66±110.5b
	Candidatus_Dormiibacterota	443.2±15.97c	486.8±9.86bc	519.57±11.55ab	561.72±41.36a	450.06±14.21c	430.79±7.94c
	Chloroflexota	345.06±15.74bc	456.73±23.48a	418.91±49.16ab	280.37±2.68c	346.01±34.08bc	273.66±21.65c
	Candidatus_Binatota	343.46±3.75bc	280.39±1.11d	314.82±16.1cd	365.33±9.27ab	396.61±11.09a	380.92±20.38ab
	Gemmatimonadota	252.75±6.15b	280.76±5.12b	284.84±6.71b	301.92±7.92b	361.58±34.18a	254.54±9.54b
	Verrucomicrobiota	225.96±7.64b	185.33±6.57b	190.45±16.67b	219.78±2.5b	252.56±16.5b	339.19±53.36a
	Methylomirabilota	172±7.36cd	140.05±6.2d	186.62±24.16bc	195.76±4.12bc	293.67±10.91a	223.55±12.53b
	Rokuibacteriota	192.13±8.38bc	155.92±12.64bc	204.23±35.08b	165.84±6.99bc	282.27±19.17a	206.6±14.85b
	others	1363.83±47.66b	1392.89±33.18b	1272.5±22.52b	1424.92±4.53b	1569.66±33.71a	1590.68±95.96a
Lignin-derived	Actinomycetota	1123.66±56.11a	897.54±23.98bc	931.24±56.96b	856.13±41.82bc	837.96±40.34bc	1085.78±61.27a
	Pseudomonadota	540.19±24.41bc	641.65±9.27a	627.56±31.41a	640.34±18.76a	591.17±9.54ab	484.54±31.03c
	Acidobacteriota	501.79±50.65b	596.55±16.63ab	536.49±34.93b	657.27±14.24a	637.51±24a	506.65±31.92b
	Binatota	146.21±9.28a	127.96±2.07a	134.05±9.41a	139.18±2.93a	150.06±5.91a	153.03±10.24a
	Dormiibacterota	112.73±6.21bc	104.05±4.27c	124.15±7.18bc	152.31±13.29a	127.1±3.89bc	113.85±2.36bc
	Gemmatimonadota	75.95±2.2b	85.96±2.47b	87.05±6.66b	80.1±5.4b	102.79±2.44a	75.9±3.84b
	Chloroflexota	57.45±7.61bc	95.67±8.37a	88.27±15.03ab	60.16±0.56bc	75.03±17.43abc	51.1±8.45c
	Rokuibacteriota	64.86±4.14b	59.24±5.16b	71.52±9.33b	58.56±5.3b	96.08±7.86a	64.03±6.81b
	Verrucomicrobiota	57.83±2.56b	59.99±3.04b	63.78±6.69ab	65.92±4.76ab	68.68±0.37ab	77.08±8.37a
	Nitrososphaerota	99.74±14.12b	19.86±2.99c	27.87±4.27c	30.51±6.84c	59.56±6.12bc	198.77±47.35a
	others	368.1±4.75cd	353.01±1.65d	365.67±10.99cd	402.95±8.71bc	451.3±15.32a	430.21±21.04ab
Chitin-derived	Actinomycetota	119.97±7.23ab	125.85±3.88a	125.06±4.11a	119.14±3.36ab	89.81±4.6c	129.99±6.29a
	Acidobacteriota	38.29±3.84b	61.71±4.2a	42.82±7.18bc	52.54±3.23ab	49.8±4.77ab	42.5±3.64b
	Pseudomonadota	23.13±0.82b	36.68±1.03a	31.83±2.3ab	33.99±4.8a	28.73±1.83ab	28.52±3.78ab
	Dormiibacterota	10.78±1.17c	17±0.8bc	25.46±2.89ab	31.58±6.08a	16.02±3.84bc	12.85±2.86c
	Saccharibacteria	11.32±0.71ab	14.41±1.96a	14.29±3.14a	12.11±0.7ab	9.41±0.86ab	7.63±0.58b
	Binatota	5.61±1.06bc	5.58±0.38bc	3.99±0.85c	12.95±1.72a	8.46±0.65b	8.85±1.24b
	Methylomirabilota	2±0.65c	5.67±1.49abc	4.79±0.65bc	8.79±1.3a	3.53±1.32c	3.47±0.22c

	Gemmatimonadota	2.75±0.67b	2.8±1.69b	3.17±1.28b	6.59±2.45ab	5.63±0.48ab	4.78±0.52ab
	Neomarinimicrobiota	2.39±1.06a	3.65±0.27a	4.91±0.7a	4.12±2.13a	2.42±0.73a	3.5±0.71a
	Thermomicrobiota	3.77±1.45a	3.56±0.69a	3.45±0.92a	2.87±0.76a	3.78±1.23a	1.85±0.25a
	others	20.52±2.65ab	25.2±1.24a	23.93±2.32a	16.07±1.94b	17.71±2ab	15.4±3.47b
Glucans-derived	Actinomycetota	41.8±1.73ab	49.63±1.29a	44.32±2.47ab	38.13±4.42bc	30.64±1.77c	38.04±2.89bc
	Pseudomonadota	20.45±1.98ab	14.98±2.5b	20.29±3.77ab	26.19±2.04a	20.82±1.77ab	24.38±1.37a
	Candidatus_Binatota	4.59±0.86ab	6.17±2.24a	5.55±0.53ab	3.97±0.47ab	2.57±0.24b	4.79±0.36ab
	Methylomirabilota	4.45±0.48a	3.71±0.6a	4.06±1.13a	2.97±0.19a	4.11±0.91a	4.42±1.06a
	Acidobacteriota	1.28±0.62a	6.06±0.85a	5.28±2.32a	3.3±1.6a	3.18±1.54a	4.27±1.54a
	Rokuibacteriota	6.14±1.74a	2.55±0.83a	2.35±0.49a	3.37±0.77a	2.73±1.6a	5.61±1.2a
	Dormiibacterota	2±0.29b	5.2±0.77a	3.35±0.5ab	1.71±1.27b	1.58±0.67b	3.32±1.07ab
	Chloroflexota	4.03±1.89a	4.2±0.88a	1.9±0.53a	2.56±0.92a	2.22±0.95a	2.56±0.49a
	Bacteroidota	0.86±0.18a	3±1.5a	1.91±0.51a	1.06±0.39a	0.71±0.37a	0.81±0.61a
	Saccharibacteria	1.19±0.43bc	0.25±0.13c	1.08±0.4bc	1.44±0.07b	0.76±0.15bc	2.7±0.38a
	others	4.99±1.69a	6.22±1.12a	6.27±0.89a	3.95±1.95a	4.15±1.38a	1.87±0.59a
Peptidoglycan-derived	Pseudomonadota	364.84±18.71cd	398.76±11.81abc	369.72±11.83bcd	444.31±21.92a	411.43±10.3abc	333.13±27.5d
	Actinomycetota	415.38±31.91a	364.83±5.24b	376.58±14.24ab	318.24±2.4c	292.18±12.73cd	372.56±12.37ab
	Acidobacteriota	178.29±19.48cd	219.42±7.45abc	185.32±15.37bcd	245.32±11.13a	229.31±10.24ab	161.25±25.14d
	Dormiibacterota	32.65±1.84bc	30.31±2.63c	38.58±2.63abc	40.62±2.44ab	32.37±3.09bc	37.01±3.23abc
	Binatota	38.53±2.13ab	38.82±2.04ab	30.38±2.69b	35.54±1.02ab	38.53±4.08ab	40.73±4.85a
	Gemmatimonadota	19.39±2.07bc	15.4±1.48c	17.6±0.67bc	21.58±2.97bc	32.27±3.03a	17.73±1.3bc
	Verrucomicrobiota	19.4±1.34bc	17.6±1.43bc	16.7±0.76bc	14.32±1.27c	21.54±0.35ab	26.57±4.34a
	Methylomirabilota	18.85±0.53abc	13.26±0.61c	20.45±3.44abc	16.32±1.33bc	21.96±2.09ab	23.69±2.14a
	Bacillota	16.67±1.42bc	18.09±1.84ab	19.93±2.35ab	21.59±1.65a	16.09±0.81bc	13.07±0.69c
	Vulcanimicrobiota	12.94±1.93c	18.76±1.93abc	16.47±2.29abc	19.66±1.85ab	12.63±1.28c	13.85±2.04bc
	others	103.17±2.73c	85.75±4.52d	108.65±6.86bc	100.26±4.79cd	131.41±4.24a	113.86±2.91bc
